# Reduced magnitude and durability of humoral immune responses to COVID-19 mRNA vaccines among older adults

**DOI:** 10.1093/infdis/jiab592

**Published:** 2021-12-09

**Authors:** Mark A Brockman, Francis Mwimanzi, Hope R Lapointe, Yurou Sang, Olga Agafitei, Peter Cheung, Siobhan Ennis, Kurtis Ng, Simran Basra, Li Yi Lim, Fatima Yaseen, Landon Young, Gisele Umviligihozo, F Harrison Omondi, Rebecca Kalikawe, Laura Burns, Chanson J Brumme, Victor Leung, Julio S G Montaner, Daniel Holmes, Mari L DeMarco, Janet Simons, Ralph Pantophlet, Masahiro Niikura, Marc G Romney, Zabrina L Brumme

**Affiliations:** 1 Faculty of Health Sciences, Simon Fraser University, Burnaby, V5A 1S6, Canada; 2 Department of Molecular Biology and Biochemistry, Simon Fraser University, Burnaby, V5A 1S6, Canada; 3 British Columbia Centre for Excellence in HIV/AIDS, Vancouver, V6Z 1Y6, Canada; 4 Department of Chemistry, Simon Fraser University, Burnaby, V5A 1S6, Canada; 5 Division of Medical Microbiology and Virology, St. Paul's Hospital, Vancouver, V6Z 1Y6, Canada; 6 Department of Medicine, University of British Columbia, Vancouver, V6T 1Z3, Canada; 7 Department of Pathology and Laboratory Medicine, Providence Health Care, Vancouver, V6Z 1Y6, Canada; 8 Department of Pathology and Laboratory Medicine, University of British Columbia, Vancouver, V6T 1Z7, Canada

**Keywords:** COVID-19, mRNA vaccine, humoral responses, older adults, antibodies, viral neutralization

## Abstract

**Background:**

The magnitude and durability of immune responses to COVID-19 mRNA vaccines remain incompletely characterized in the elderly.

**Methods:**

Anti-spike RBD antibodies, ACE2 competition and virus neutralizing activities were assessed in plasma from 151 healthcare workers and older adults (range 24-98 years of age) one month following the first vaccine dose, and one and three months following the second dose.

**Results:**

Older adults exhibited significantly weaker responses than younger healthcare workers for all humoral measures evaluated and at all time points tested, except for ACE2 competition activity after one vaccine dose. Moreover, older age remained independently associated with weaker responses even after correction for sociodemographic factors, chronic health condition burden, and vaccine-related variables. By three months after the second dose, all humoral responses had declined significantly in all participants, and remained significantly lower among older adults, who also displayed reduced binding antibodies and ACE2 competition activity towards the Delta variant.

**Conclusions:**

Humoral responses to COVID-19 mRNA vaccines are significantly weaker in older adults, and antibody-mediated activities in plasma decline universally over time. Older adults may thus remain at elevated risk of infection despite vaccination.

**Funding statement:** This work was supported by the Public Health Agency of Canada through a COVID-19 Immunology Task Force COVID-19 "Hot Spots" Award (2021-HQ-000120 to MAB, ZLB, MGR) and the Canada Foundation for Innovation through Exceptional Opportunities Fund – COVID-19 awards (to MAB, MN, MLD, RP, ZLB) and the National Institute of Allergy and Infectious Diseases of the National Institutes of Health (R01AI134229 to RP). GU and FHO are supported by Ph.D. fellowships from the Sub-Saharan African Network for TB/HIV Research Excellence (SANTHE), a DELTAS Africa Initiative (grant # DEL-15-006). The DELTAS Africa Initiative is an independent funding scheme of the African Academy of Sciences (AAS)'s Alliance for Accelerating Excellence in Science in Africa (AESA) and supported by the New Partnership for Africa's Development Planning and Coordinating Agency (NEPAD Agency) with funding from the Wellcome Trust (grant # 107752/Z/15/Z) and the UK government. The views expressed in this publication are those of the authors and not necessarily those of AAS, NEPAD Agency, Wellcome Trust or the UK government. LYL was supported by an SFU Undergraduate Research Award. MLD and ZLB hold Scholar Awards from the Michael Smith Foundation for Health Research.

## INTRODUCTION

Older age is the strongest and most common risk factor for lethal coronavirus disease 2019 (COVID-19) following severe acute respiratory syndrome coronavirus 2 (SARS-CoV-2) infection [1-3]. While COVID-19 vaccines offer hope to end the pandemic [4-7], real world assessments have revealed weaker vaccine-induced immune responses in certain groups including the elderly [8-16], though few studies have adjusted for potential confounders, including comorbidities, that can accumulate with age. Vaccine response durability and cross-reactivity towards SARS-CoV-2 variants of concern also remain incompletely characterized, as immunogenicity assessments are occurring concomitantly with national vaccine rollouts and emergence of new strains.

Two mRNA vaccines, Comirnaty (BNT162b2; Pfizer/BioNTech) and Spikevax (mRNA-1273; Moderna), have been administered widely. Both vaccines provided >94% protection against moderate or severe COVID-19 in clinical trials after two doses [6, 7] and population-level reductions in COVID-19 were observed following initial vaccine rollouts, but ongoing outbreaks in long-term care facilities underscore the continuing vulnerability of older adults to SARS-CoV-2, even after vaccination [14, 17, 18]. Age and age-associated comorbidities, including chronic health conditions that result in immune dysregulation, have been linked to poor vaccine responses [19-21], but few studies have explored these variables in the context of COVID-19 immunization. A better understanding of the impact of age and age-related factors on the magnitude and durability of vaccine-induced immune responses can inform public health decision-making around COVID-19 vaccine allocation as the pandemic progresses.

We investigated the magnitude of SARS-CoV-2 spike-specific humoral immune responses in plasma one month after the first mRNA vaccine dose, and one and three months following the second dose in 151 participants aged 24-98 years. We also assessed responses against the widely circulating Delta variant (B.1.617.2) at one and three months following the second vaccine dose. Our results demonstrate weaker humoral responses to COVID-19 mRNA vaccines in older versus younger adults, signified by reduced magnitude and durability of spike-specific binding antibodies, ACE2 competition activity and neutralizing antibody activity even after correction for potential confounders. Reduced humoral responses were also observed against the Delta variant, indicating that older adults may remain at higher risk of infection by this predominant circulating strain despite vaccination.

## METHODS

***Study design.*** We conducted a prospective longitudinal cohort study in British Columbia, Canada, to examine SARS-CoV-2 specific humoral immune responses following vaccination with COVID-19 mRNA vaccines Comirnaty (Pfizer/BioNTech BNT162b2) or Spikevax (Moderna mRNA-1273). Our cohort of 151 individuals included 89 healthcare workers and 62 older adults (comprising 23 residents of long-term care or assisted living facilities and 39 seniors living independently).

***Ethics approval.*** Written informed consent was obtained from all participants or their authorized decision makers. This study was approved by the University of British Columbia/Providence Health Care and Simon Fraser University Research Ethics Boards.

***Participants and sampling.*** Participants were recruited at facilities operated by Providence Health Care (Vancouver, Canada) and from the community. Serum and plasma were collected prior to vaccination, at one month after the first dose, and at one and three months after the second dose. Specimens were processed same-day and frozen until analysis.

***Data sources and immune measures.*** Sociodemographic data (age, sex, ethnicity), chronic health conditions and COVID-19 vaccination information were collected by self-report and confirmed through medical records where available. Chronic health conditions were defined as hypertension, diabetes, asthma, obesity (defined as having a body mass index ≥30), chronic diseases of lung, liver, kidney, heart or blood, cancer, and immunosuppression due to chronic conditions or medication, to generate a total score ranging from 0-11 per participant. Vaccine-induced responses were assessed using (1) a commercial assay to detect IgG antibodies targeting the spike receptor binding domain (RBD); (2) a commercial angiotensin-converting enzyme 2 (ACE2) competition assay to detect antibodies that block RBD-receptor interaction; and (3) virus neutralization assays to detect antibodies that prevent SARS-CoV-2 infection of target cells.

***Binding antibody assays.*** COVID-19 convalescent individuals were identified by the presence of serum antibodies recognizing SARS-CoV-2 nucleoprotein (N) using the Elecsys Anti-SARS-CoV-2 assay on a Cobas e601 module analyzer (Roche Diagnostics). Plasma IgG binding antibodies against RBD were quantified using ELISA (V-plex SARS-CoV-2; Meso Scale Diagnostics) on a Meso QuickPlex SQ120 instrument as directed by the manufacturer. Results were calibrated against a WHO-referenced standard and are report as international binding antibody units (BAU)/mL.

***ACE2 competition assay***. The ability of plasma antibodies to block the RBD-ACE2 receptor interaction was assessed by competition ELISA (V-plex SARS-CoV-2; Meso Scale Diagnostics) on a Meso QuickPlex SQ120 instrument as directed by the manufacturer. Results were calibrated against an external standard and are reported as arbitrary units (AU)/mL, with an upper limit of quantification of 35 (or 1.54 $\log_2$) AU/mL.

***Virus neutralization assays***. Neutralizing activity in plasma was examined using a live SARS-CoV-2 infectivity assay at Containment Level 3. Assays were performed using isolate USA-WA1/2020 (BEI Resources) and VeroE6-TMPRSS2 (JCRB-1819) target cells. Virus stock was adjusted to 50 $TCID_{50}$/200 µl in Dulbecco's Modified Eagle Medium in the presence of serial 2-fold dilutions of plasma (1:20 to a maximum of 1:2560), incubated at 4°C for 1 hour, then added to target cells in 96-well plates in triplicate and incubated at 37°C with 5% $CO_2$. Viral cytopathic effect (CPE) was recorded on day 3 post-infection. Neutralizing activity is reported as "present" if CPE was prevented in all three wells at a 1:20 dilution (binary variable); or as the reciprocal plasma dilution necessary to prevent CPE in all three wells (continuous variable). After one dose of vaccine, neutralizing activity was reported as "borderline" if CPE was prevented in any of three wells at a 1:20 dilution.

***Statistical analysis.*** Comparisons of binary variables were performed using Fisher's exact test. Comparisons of continuous variables were performed using the Mann-Whitney U-test (for unpaired data) or Wilcoxon test (for paired data). Ordinary least squares regression was used to examine relationships between continuous variables. Multiple linear regression was employed to investigate the relationship between age (per year increment), sex (female as reference group), Ethnicity (non-white as reference group), number of chronic health conditions (per number increment), vaccine type (Comirnaty as reference group), dosing interval (per day increment) and sampling date following vaccine dose (per day increment) on immunogenicity outcomes. All tests were two-tailed, with p=0.05 considered statistically significant. Analyses were conducted using Prism v9.3.0 (GraphPad).

## RESULTS

***Lower RBD binding antibodies associated with older age and chronic health conditions.***

Characteristics of the 151 participants, which included 89 healthcare workers (HCW) and 62 older adults, are shown in **Table 1**. All participants received two doses of mRNA vaccine between December 2020 and July 2021. Due to limited initial vaccine supply in British Columbia, the interval between doses was extended to a maximum of 112 days on March 1, 2021, so participants received their second dose a median of 91 days after the first (interquartile range [IQR] 70-99 days). Samples were collected before vaccination to assess prior exposure to SARS-CoV-2 (n=142); at one month following the first (n=141) and second (n=150) doses to quantify response magnitude; and at three months following the second dose (n=150) to examine response durability.

As shown in **Table 1**, HCW and older adults were a median of 41 and 79 years old respectively, and predominantly female. At entry, 14 participants (9.3%; eight HCW and six older adults) were identified as COVID-19 convalescent based on the presence of anti-SARS-CoV-2 N antibodies. Nine participants (6%; one HCW and eight older adults) received Spikevax for their first dose, while 142 (94%) received Comirnaty. In addition to age, the groups differed significantly in terms of ethnicity (p=0.0002), number of chronic health conditions (p<0.0001, where the two most common conditions were hypertension and diabetes), vaccine received (p=0.0015) and time between doses (p<0.0001). The groups also differed in terms of the exact day of specimen collection after the first dose (p=0.0069) and at three months after the second dose (p=0.011), though these differences were one day or less.

We quantified anti-RBD IgG binding antibodies in plasma one month after the first and second vaccine doses using ELISA, where the latter time point should capture peak immunity. After one dose, median anti-RBD IgG concentrations were 2.5-fold lower in older adults who were naïve to COVID-19, compared to COVID-19 naïve HCW (p<0.0001) (**Figure 1A**). In contrast, COVID-19 convalescent participants mounted ~17- and ~42-fold higher IgG responses after one dose compared to COVID-19 naïve HCW and older adults, respectively (both p<0.0001), consistent with prior studies demonstrating robust reactivity to one dose in previously infected individuals [22, 23]. After two doses, median anti-RBD IgG concentrations increased by ~10-fold in both naïve groups, but responses remained two-fold lower among older adults (p<0.0001) (**Figure 1C**). No further increase in IgG antibodies was observed in convalescent participants. Indeed, after two doses the median IgG values in HCW reached equivalence with the convalescent group, while values in older adults remained 1.7-fold lower (p<0.0001) (**Figure 1C**). Of note, one doubly vaccinated older adult continued to exhibit a very poor response (**Figure 1C**).

Among COVID-19 naïve individuals, we estimated using univariable linear regression that every decade of older age was associated, on average, with 0.14 and 0.09 $\log_{10}$ lower IgG responses one month after one and two vaccine doses, respectively (both p<0.0001) (**Figures 1B,D**). Multivariable analyses adjusting for sex, ethnicity, number of chronic health conditions, vaccine brand, dosing interval and day of specimen collection post-immunization confirmed that older age remained significantly negatively associated with IgG responses one month after one and two vaccine doses (p=0.0001 and p=0.0002, respectively). A higher number of chronic health conditions was also negatively associated with IgG responses after one dose (p=0.03) (**Table 2**).

***Reduced ability to block ACE2 binding associated with older age and male sex.***

We next assessed the ability of plasma antibodies to block the interaction between RBD and ACE2 receptor using competition ELISA, which offers a surrogate measure of virus neutralizing activity [24]. After one vaccine dose, HCW and older adults exhibited median ACE2 competition activities of 2.8 (or 0.45 $\log_{10}$) and 2.5 (or 0.40 $\log_{10}$) AU/mL, respectively, a difference that was not statistically significant (**Figure 2A**). In contrast, after one dose most (10, or 77%) convalescent participants exhibited a median activity above the upper limit of quantification (ULOQ) for this assay (35, or 1.54 $log_{10}$ AU/mL) ($p<0.0001$ compared to both naive groups). One month following the second vaccine dose, HCW exhibited a median activity of 15 (or 1.2 $log_{10}$) AU/mL compared to 6.7 (or 0.82 $log_{10}$) AU/mL in older adults (p=0.0002) (**Figure 2C**), with 26 (44%) HCW and 6 (11%) older adults above the ULOQ.  Meanwhile, convalescent participants maintained a median activity of 35 (1.54 $log_{10}$) AU/mL after the second dose, with 8 (57%) individuals exceeding the ULOQ (p=0.0006 compared to older adults). These results are consistent with other studies showing that qualitative features of antibody function including virus neutralizing activity may be enhanced following infection compared to vaccination [25, 26], and further suggest that these features may be diminished with older age.

Even though ACE2 competition activities were not significantly different between HCW and older adults following one vaccine dose, age-related effects were apparent when age was analyzed as a continuous variable in all COVID-19 naïve participants. Specifically, we estimated using univariable linear regression that every 10 years of older age was associated with 0.021 and 0.071 $log_{10}$ AU/mL lower ACE2 competition activity (equivalent to 1.0 and 1.2 AU/mL) one month after the first and second doses, respectively (p=0.03 and <0.0001) (**Figures 2B,D**). Multivariable analyses confirmed that age remained negatively associated with ACE2 competition activity one month after the second dose (p=0.02) (**Table 2**).  Female sex was independently associated with 0.094 $log_{10}$ (or 1.24) AU/ml higher ACE2 competition activity after the first dose (p=0.03), which is consistent with reports that females display higher neutralizing responses following infection and vaccination [27].

***Weaker virus neutralizing activity associated with age and vaccine product.***

We next performed live SARS-CoV-2 neutralization assays to quantify the ability of plasma to block infection of target cells, which may involve spike epitopes located outside the RBD [28, 29]. As neutralization activities following one vaccine dose were generally weak in COVID-19 naïve individuals, for this timepoint we considered both "clear positive" samples that neutralized virus in all three wells and "borderline" samples that neutralized virus in at least one well at a 1:20 dilution. Using this latter definition, 16/78 (21%) HCW and 2/44 (4.8%) older adults displayed evidence of neutralizing activity (p=0.02; **Figure 3A**). In contrast, plasma from all 13 convalescent participants neutralized SARS-CoV-2 following one vaccine dose (median reciprocal titer of 240). One month following the second vaccine dose and using the more stringent definition of "clear positive", 79/81 (98%) HCW displayed neutralizing activity compared to 44/52 (85%) older adults (p=0.01; **Figure 3C**).

Among COVID-19 naïve individuals, univariable linear regression confirmed a statistically significant inverse relationship between virus neutralization activity and older age following one and two vaccine doses (p=0.003 and p<0.0001, respectively) (**Figures 3B,D**). In multivariable analyses, older age remained significantly associated with weaker neutralization activity after both one and two doses (p=0.002 and p=0.0002, respectively) (**Table 2**). Having received Spikevax was also associated with stronger neutralization activity following one dose (p=0.04). Notably, ACE2 competition activity correlated with virus neutralizing activity after the second dose (Spearman $\rho \geq 0.7$; all p<0.0001) (**Supplemental Figure 1**).

***Vaccine-induced antibody responses decline over time in all ages***

To examine immune response durability, we reassessed humoral outcomes three months following the second vaccine dose. All three measures of antibody activity declined between one and three months following the second immunization: median IgG binding antibodies declined 2-fold in both HCW and older adults (Wilcoxon paired test, both p<0.0001; **Figure 4A**), while median ACE2 competition activity declined by 2.6-fold in HCW and by 1.7-fold in older adults (Wilcoxon, both p<0.0001) (**Figure 4B**). Furthermore, median virus neutralizing activity declined 4-fold in HCW (p<0.0001) and 2-fold in older adults (Wilcoxon, p<0.0001) (**Figure 4C**). Despite these temporal reductions, responses in HCW remained significantly higher compared to older adults in all assays at three months after the second dose (**Supplemental Figure 2)**, and age remained a significant independent predictor of reduced activity for all measures in both univariable and multivariable analyses (**Supplemental Figure 2** and **Table 2**). For context, the median residual activities observed in HCW at three months after the second dose were comparable to peak responses seen in older adults at one month after this dose.

***Impaired ability to block ACE2 binding by Delta variant among older adults.***

Given concerns that SARS-CoV-2 variants may be more transmissible or evade aspects of host immunity [30-32], we examined IgG binding antibodies and ACE2 competition activity against the B.1.617.2 (Delta) variant at one and three months following the second vaccine dose. Consistent with our observations for the original Wuhan strain, binding antibody responses to the Delta RBD were ~2-fold lower among older adults compared to HCW at both timepoints (Mann-Whitney, both $p<0.0001$) (**Figure 5A**). Within each group however, median binding antibody values were broadly comparable between the two strains at each timepoint tested, where within-group comparisons were either not significantly different or only modestly lower despite achieving statistical significance (*e.g.* differences $<0.02$ $\log_{10}$ for HCW and older adults; $<0.08$ $\log_{10}$ for convalescents). ACE2 competition activity against Delta RBD was also significantly lower among older adults compared to HCW at one and three months after the second dose (Mann-Whitney, both $p<0.0001$) (**Figure 5B**). Moreover, plasma specimens from all groups consistently displayed significantly weaker ability to block ACE2 receptor engagement by the Delta RBD compared to that of Wuhan RBD (Wilcoxon, all $p\leq0.01$), though the magnitude of these differences was modest (~0.04 $\log_{10}$ in HCW, ~0.03-0.06 $\log_{10}$ in older adults).

## DISCUSSION

This study extends our understanding of antibody response magnitude and durability following COVID-19 mRNA vaccination across the adult age spectrum [14, 18, 33-36]. Overall, responses in older adults are impaired both quantitatively (i.e., fewer binding antibodies) and functionally (i.e., lower ACE2 displacement and neutralization activities) compared to younger adults, even after two vaccine doses. Importantly, multivariable analyses confirmed older age as an independent determinant of poorer immune responses at nearly all timepoints evaluated following both one and two vaccine doses, even after controlling for chronic health conditions that can accumulate with age and compromise immunity [19-21]. The sole exception was ACE2 competition activity one month after the first dose, which did not remain independently associated with age after multivariable correction. Multivariable analyses identified additional correlates of humoral responses following the first vaccine dose, including the number of chronic health conditions (associated with lower binding antibody titers), male sex (associated with lower ACE2 competition activity), and having received Spikevax (associated with higher virus neutralizing activity). In general, the impact of these variables on humoral responses diminished after the second vaccine dose, though the number of chronic conditions was again associated with poorer binding antibody responses three months after the second dose. Our results thus identify age as the most critical and consistent variable modulating the magnitude of antibody responses after COVID-19 mRNA vaccination.

Our findings also shed light on the short-term durability of humoral responses to COVID-19 mRNA vaccines. By three months following the second vaccine dose, plasma antibody concentrations had declined significantly in all participants, particularly those who were naive to COVID-19 prior to vaccination. Assuming exponential decay, we estimate the half-life of anti-RBD binding antibodies to be 87 days [95% CI 75-97] in the naïve group, which suggests that antibody durability following mRNA vaccination may be lower compared to that following infection, which was calculated to be ~116 days in a study of convalescent individuals [37]. More importantly, humoral responses remained substantially lower among older adults at all timepoints tested. For context, the "diminished" responses observed in HCW at three months following the second vaccine dose were comparable to peak levels observed in older adults at one month following the second dose. Similar results for antibody binding and ACE2 competition activity were found for the B.1.617.2 (Delta) variant RBD, suggesting that older adults will remain more susceptible to infection by this variant at all stages after vaccination due to their weaker overall responses.

Our observations are consistent with poorer immune responses to certain immunizations (*e.g.* influenza) among older adults that can be mitigated in part by modifying vaccine formulations (*e.g.*, by increasing antigen concentrations or additional adjuvants) or providing booster immunizations more frequently [19-21]. Reports from the UK [15] and Germany [16] have also demonstrated age-related impairments in binding and neutralizing antibodies following immunization with the Comirnaty vaccine, though T cell responses were more similar between younger and older participants. However, these studies did not examine the durability of vaccine-induced immune responses in older adults, which is of paramount importance as more time elapses after people complete the standard two-dose vaccine schedule. Indeed, recent increases in SARS-CoV-2 infections among doubly vaccinated individuals [38], including outbreaks in long-term care facilities [17], underscore this ongoing risk.

Our findings that 14% of older adults failed to neutralize SARS-CoV-2 (USA-WA1/2020 strain) one month after having received two vaccine doses, a timepoint that should capture the "peak" vaccine immune response, and that this percentage increased to 44% just two months later, further emphasizes the ongoing infection risk in this population. While we did not perform virus neutralization assays using the Delta variant, our ACE2 competition results using the RBD of this strain suggest that neutralization activity against Delta is likely to be lower than that against the Wuhan strain. Given the ability of SARS-CoV-2 variants to evade at least some aspects of vaccine-elicited immunity [39, 40], our results support ongoing prioritization of older adults for receipt of additional vaccine doses.

A limitation of our study is that immune correlates of protection for SARS-CoV-2 transmission and disease severity remain incompletely characterized [41], so the implications of our results as they relate to individual-level control of COVID-19 remain uncertain. Since precise antibody concentrations and activities needed to achieve protection are unknown, it is possible that the vaccine-induced immune responses seen in older adults will be sufficient to prevent symptomatic infection or severe disease in many cases. Additional studies linking vaccine immunogenicity data to clinical outcomes specifically among older adults are needed. In addition, we have not assessed the cellular immune responses induced by vaccination. Antiviral T cell responses are durable following infection and immunization with mRNA vaccines [42-47] but more research is needed to define their role in long-term protection against infection and disease. Furthermore, due to the small number of participants who received Spikevax, we had low power to assess differences in responses between mRNA vaccines. Nevertheless, and consistent with recent studies [17, 48], Spikevax was associated with improved virus neutralization activity following a single vaccine dose in our analysis.

Overall, our results extend a growing body of evidence indicating that COVID-19 mRNA vaccines are less immunogenic in older adults and further reveal substantial declines of humoral responses in plasma across all ages in the first three months following completion of a two-dose vaccine series. The combined effects of lower peak immunity and natural declines in vaccine-induced humoral responses may leave older adults at continued risk of infection by SARS-CoV-2 or its variants.

**FUNDING**

This work was supported by the Public Health Agency of Canada through a COVID-19 Immunology Task Force COVID-19 "Hot Spots" Award (2021-HQ-000120 to MAB, ZLB, MGR) and the Canada Foundation for Innovation through Exceptional Opportunities Fund – COVID-19 awards (to MAB, MN, MLD, RP, ZLB) and the National Institute of Allergy and Infectious Diseases of the National Institutes of Health (R01AI134229 to RP). GU and FHO are supported by Ph.D. fellowships from the Sub-Saharan African Network for TB/HIV Research Excellence (SANTHE), a DELTAS Africa Initiative [grant # DEL-15-006]. The DELTAS Africa Initiative is an independent funding scheme of the African Academy of Sciences (AAS)'s Alliance for Accelerating Excellence in Science in Africa (AESA) and supported by the New Partnership for Africa's Development Planning and Coordinating Agency (NEPAD Agency) with funding from the Wellcome Trust [grant # 107752/Z/15/Z] and the UK government. The views expressed in this publication are those of the authors and not necessarily those of AAS, NEPAD Agency, Wellcome Trust or the UK government. LYL was supported by an SFU Undergraduate Research Award. MLD and ZLB hold Scholar Awards from the Michael Smith Foundation for Health Research.

**ACKNOWLEDGEMENTS**

We thank the leadership and staff of Providence Health Care, including long-term care and assisted living residences, for their support of this study. We thank the phlebotomists and laboratory staff at St. Paul's Hospital, the BC Centre for Excellence in HIV/AIDS and Simon Fraser University for assistance. Above all, we thank the participants, without whom this study would not have been possible.

## Tables

**Table 1. Study Participants (n=151)**

| | **Healthcare Workers (n=89)** | **Older Adults (n=62)** | **p-value** |
|---|---|---|---|
| Age in years, median [IQR][a] | 41 [35-50] | 79 [73-86] | **<0.0001** |
| Female Sex, n (%) | 65 (73%) | 43 (69%) | 0.71 |
| White/Caucasian Ethnicity, n (%) | 40 (45%) | 47 (76%) | **0.0002** |
| COVID-19 convalescent (anti-N Ab+), n (%) | 8 (9%) | 6 (10%) | >0.99 |
| Chronic health or immunosuppresive conditions, median [IQR] | 0 [0-0] | 1 [0-2] | **<0.0001** |
| Comirnaty mRNA Vaccine, n (%) | 88 (99%) | 54 (87%) | **0.0015** |
| Time between doses in days, median [IQR] | 97 [91-103] | 78 [45-86] | **<0.0001** |
| Specimens collected one month after first dose, n (%) | 87 (98%) | 54 (87%) | n.a.[b] |
| Day of specimen collection one month after first dose, median [IQR] | 29 [27-31] | 30 [28-32] | **0.0069** |
| Specimens collected one month after second dose, n (%) | 89 (100%) | 61 (98%) | n.a. |
| Day of specimen collection one month after second dose, median [IQR] | 30 [29-32] | 30 [29-32] | 0.78 |
| Specimens collected three months after second dose, n (%) | 89 (100%) | 61 (98%) | n.a. |
| Day of specimen collection three months after second dose, median [IQR] | 90 [90-91] | 90 [88-91] | **0.011** |

[a] IQR, interquartile range; [b] n.a., not applicable

**Table 2. Multivariable Analyses**

| Immunogenicity outcome | Variable | Time point: 1 month after 1st dose β estimate (95% CI [a]) | p | Time point: 1 month after 2nd dose β estimate (95% CI) | p | Time point: 3 months after 2nd dose β estimate (95% CI) | p |
|---|---|---|---|---|---|---|---|
| RBD IgG (log10 international units) | Age (per year) | -0.011 (-0.017 to -0.0055) | **0.0001** | -0.0090 (-0.014 to -0.0044) | **0.0002** | -0.0080 (-0.012 to -0.0039) | **0.0002** |
| | Male Sex | -0.068 (-0.25 to 0.12) | 0.5 | -0.11 (-0.27 to 0.053) | 0.2 | 0.044 (-0.097 to 0.18) | 0.5 |
| | White ethnicity | -0.062 (-0.25 to 0.12) | 0.5 | 0.063 (-0.095 to 0.22) | 0.4 | 0.17 (0.031 to 0.31) | **0.02** |
| | # Chronic conditions (per additional) | -0.10 (-0.19 to -0.0073) | **0.03** | -0.047 (-0.12 to 0.028) | 0.2 | -0.070 (-0.14 to -0.0038) | **0.04** |
| | SpikeVax vaccine | 0.26 (-0.83 to 0.60) | 0.1 | 0.20 (-0.059 to 0.46) | 0.1 | 0.15 (-0.088 to 0.39) | 0.2 |
| | Sampling date[b] (per day) | -0.012 (-0.047 to 0.023) | 0.5 | 0.0040 (-0.027 to 0.035) | 0.8 | 0.0036 (-0.021 to 0.028) | 0.8 |
| | Dosing interval[c] (per day) | n.a.[d] | - | -0.0016 (-0.0050 to 0.0017) | 0.3 | -0.0017 (-0.0046 to 0.0013) | 0.3 |
| ACE2 competition (log10 units) | Age (per year) | -0.0016 (-0.0040 to 0.00088) | 0.2 | -0.0053 (-0.0096 to -0.00093) | **0.02** | -0.0039 (-0.0071 to -0.00069) | **0.02** |
| | Male Sex | -0.094 (-0.18 to -0.011) | **0.03** | -0.068 (-0.22 to 0.082) | 0.4 | 0.019 (-0.095 to 0.13) | 0.7 |
| | White ethnicity | -0.046 (-0.13 to 0.035) | 0.3 | -0.098 (-0.25 to 0.051) | 0.2 | 0.064 (-0.047 to 0.17) | 0.3 |
| | # Chronic conditions (per additional) | -0.015 (-0.056 to 0.026) | 0.5 | -0.029 (-0.10 to 0.042) | 0.4 | -0.028 (-0.081 to 0.026) | 0.3 |
| | SpikeVax vaccine | 0.098 (-0.052 to 0.25) | 0.2 | 0.12 (-0.12 to 0.37) | 0.3 | 0.15 (-0.049 to 0.34) | 0.1 |
| | Sampling date[b] (per day) | 0.0064 (-0.0091 to 0.022) | 0.4 | -0.0070 (-0.036 to 0.022) | 0.6 | -0.000058 (-0.0023 to 0.0024) | 1 |
| | Dosing interval[c] (per day) | n.a. | - | 0.0019 (-0.0012 to 0.050) | 0.2 | -0.0069 (-0.027 to 0.013) | 0.5 |
| Viral neutralization (log2 reciprocal dilution) | Age (per year) | -0.0080 (-0.013 to -0.0030) | **0.002** | -0.032 (-0.048 to -0.016) | **0.0002** | -0.025 (-0.040 to -0.0095) | **0.002** |
| | Male Sex | -0.079 (-0.25 to 0.090) | 0.4 | -0.36 (-0.92 to 0.21) | 0.2 | 0.11 (-0.43 to 0.65) | 0.7 |
| | White ethnicity | 0.0078 (-0.16 to 0.17) | 0.9 | -0.14 (-0.70 to 0.42) | 0.6 | 0.25 (-0.28 to 0.78) | 0.3 |
| | # Chronic conditions (per additional) | 0.049 (-0.034 to 0.13) | 0.2 | -0.056 (-0.32 to 0.21) | 0.7 | -0.16 (-0.41 to 0.097) | 0.2 |
| | SpikeVax vaccine | 0.40 (0.014 to 0.79) | **0.04** | 0.75 (-0.17 to 1.67) | 0.1 | 0.38 (-0.54 to 1.32) | 0.4 |
| | Sampling date[b] (per day) | 0.0077 (-0.024 to 0.039) | 0.6 | 0.022 (-0.088 to 0.13) | 0.7 | -0.071 (-0.17 to 0.023) | 0.1 |
| | Dosing interval[c] (per day) | n.a. | - | 0.0024 (-0.0094 to 0.014) | 0.4 | -0.00088 (-0.012 to 0.010) | 0.9 |

[a] CI, confidence interval; [b] day of specimen collection following last dose; [c] days elapsed between first and second vaccine dose (where applicable); [d] n.a., not applicable

**Figure Legends**

**Figure 1: Vaccine-induced binding antibody responses to spike are lower in older adults.** *Panel A*: Binding IgG responses to the SARS-CoV-2 spike RBD in plasma, measured by ELISA, following one dose of a COVID-19 mRNA vaccine, are shown for healthcare workers (blue circles) and older adults (orange circles) who were COVID-19 naive at study entry. The third group, "N Sero+" (grey circles), denotes convalescent participants with anti-N antibodies at study entry. Values are reported in $\log_{10}$ international binding antibody units (BAU)/mL. Red bars and whiskers represent median and IQR, with group medians shown below. P-values computed using the Mann-Whitney U-test are shown above each comparison. *Panel B*: Same data from the COVID-19 naive participants shown in panel A, but plotted by age, and where participants are colored based on their number of chronic health conditions, which remained significant in multivariable analyses (see Table 2). Statistics were computed using ordinary least-squares regression, also shown as red dashed line. *Panels C, D:* Same as A and B, but for responses measured one month following two doses of mRNA vaccine.

**Figure 2: Vaccine-induced ACE2 receptor competition activity is weaker in older adults.** *Panel A*: Ability of vaccine-induced plasma antibodies to displace soluble ACE2 receptor from spike RBD, measured by ELISA, following one dose of vaccine in COVID-19 naive healthcare workers (blue circles) and older adults (orange circles), as well as COVID-19 convalescent participants ("N Sero+"; grey circles). Values represent arbitrary units (AU)/ml calibrated against an external standard, reported in $\log_{10}$ units. Red bars and whiskers represent median and IQR, with group medians shown below. P-values computed using the Mann-Whitney U-test are shown above each comparison. ULOQ, upper limit of quantification. *Panel B*: Same data from the COVID-19 naive participants shown in panel A, but plotted by age, and colored by sex, which remained significant in multivariable analyses following one dose (see Table 2). Statistics were computed using ordinary least-squares regression, also shown as red dashed line. *Panels*

*C, D:* Same as A and B, but for responses measured one month following two doses of mRNA vaccine.

**Figure 3: Viral neutralization activity of vaccine-induced antibodies is weaker in older adults.** *Panel A*: Frequency of COVID-19 naive healthcare workers (blue) and older adults (orange), as well as convalescent participants ("N Sero+"; black) are shown as histograms. The proportion of participants in each group who displayed neutralizing activity against live SARS-CoV-2 (USA-WA1/2020 strain) in 3/3 wells at a 1:20 or higher plasma dilution is shown in the darkest color, while those displaying "borderline" activity (defined as neutralization in least one well at a 1:20 dilution) is shown in lighter color. Total Ns in each group are shown above each bar, with the N of participants displaying each activity shown within the bar. P-values computed using Fisher's exact test are shown above each comparison. *Panel B*: Same data as the COVID-19 participants shown in panel A (where neutralization includes both "yes" and "borderline" categories), but plotted by age, and where neutralization is reported as the reciprocal $\log_2$ dilution value. Samples that displayed no evidence of neutralization were coded as having a reciprocal dilution factor of 10 (3.32 $\log_2$). Symbols are colored by vaccine received, which was significantly associated with neutralization activity after one dose (see Table 2). Statistics were computed using ordinary least-squares regression. LLOQ, lower limit of quantification. *Panels C, D*: Same as A and B, but for neutralization responses measured one month after two doses of mRNA vaccine and where neutralizing activity required inhibition of cytopathic effects in 3/3 wells at a 1:20 or higher plasma dilution.

**Figure 4: Older adults display lower magnitude and durability of antibody responses after vaccination.** *Panel A:* Binding IgG responses to spike RBD, measured by ELISA, one month after the first vaccine dose ($1^{st}$, light green box), one month after the second vaccine dose ($2^{nd}$, green box) and three months after this dose (3m, red box) are shown for healthcare workers (HCW; blue labels) and older adults (Older; orange) who were COVID-19 naive at study entry, and convalescent participants (N Sero+). Values are reported in $\log_{10}$ international binding antibody units (BAU)/mL. Median values are displayed below each group. P-values (computed using the Wilcoxon paired test for within-group comparisons, and the Mann-Whitney U-test for between-group comparisons) are shown above each comparison performed. The N of pairs compared in the Wilcoxon paired test is shown below the relevant time points. *Panel B*: ACE2 competition assay results in the same individuals, measured by ELISA. Values represent arbitrary units (AU)/ml calibrated against an external standard, reported in $\log_{10}$ units. ULOQ, upper limit of quantification. *Panel C*: Virus neutralization assay results in the same individuals, displayed as the reciprocal $\log_2$ plasma dilution. Note that some values are superimposed. LLOQ, lower limit of quantification.

**Figure 5: Cross-reactive antibody responses against the Delta variant after two doses of mRNA vaccine.** *Panel A:* Binding IgG antibody responses to spike RBD from the original Wuhan (Wuh) strain and the Delta (Delt) variant, measured by ELISA, one month following the second vaccine dose (peak, green box) and three months after this dose (durability, red box) in healthcare workers (HCW; blue) and older adults (Older; orange) who were COVID-19 naive at study entry, and convalescent participants (N Sero+; grey). Values reported in $\log_{10}$ international binding antibody units (BAU)/mL. Median values are shown below each group. P-values (computed using the Wilcoxon paired test for within-group comparisons, and the Mann-Whitney U-test for between-group comparisons) are shown above each comparison performed). The N of pairs compared in the Wilcoxon paired test is shown below the relevant time points. *Panel B*: ACE2 competition results in the same individuals, measured by ELISA. Values represent arbitrary units (AU)/ml calibrated against an external standard, reported in $\log_{10}$ units. ULOQ, upper limit of quantification.

Figure 1

Figure 2

Figure 3

Figure 4

Figure 5

A
IgG RBD (BAU/mL, log10)
<0.0001
<0.0001
ns
0.002
0.0006
0.002
ns
0.0002
2.8
2.8
2.5
2.5
2.8
2.7
2.5
2.5
2.2
2.2
2.7
2.5
Wuh
Delt
81
55
14
79
50
13
n (pairs)
HCW
Older
N Sero+
1 mo post 2nd dose
3 mo post 2nd dose
B
ACE2 competition (AU/mL, log10)
ULOQ
<0.0001
<0.0001
<0.0001
<0.0001
0.002
<0.0001
0.01
0.0007
1.2
1.1
0.8
0.8
1.5
1.2
0.8
0.7
0.6
0.6
0.9
0.8

## Supplementary Material

jiab592_suppl_Supplementary_Figure_S1

jiab592_suppl_Supplementary_Figure_S2

