# Reduced Magnitude and Durability of Humoral Immune Responses to COVID-19 mRNA Vaccines Among Older Adults

**DOI:** 10.1093/infdis/jiab592

**Published:** 2021-12-09

**Authors:** Mark A Brockman, Francis Mwimanzi, Hope R Lapointe, Yurou Sang, Olga Agafitei, Peter K Cheung, Siobhan Ennis, Kurtis Ng, Simran Basra, Li Yi Lim, Fatima Yaseen, Landon Young, Gisele Umviligihozo, F Harrison Omondi, Rebecca Kalikawe, Laura Burns, Chanson J Brumme, Victor Leung, Julio S G Montaner, Daniel Holmes, Mari L DeMarco, Janet Simons, Ralph Pantophlet, Masahiro Niikura, Marc G Romney, Zabrina L Brumme

**Affiliations:** 1 Faculty of Health Sciences, Simon Fraser University, Burnaby, Canada; 2 Department of Molecular Biology and Biochemistry, Simon Fraser University, Burnaby, Canada; 3 British Columbia Centre for Excellence in HIV/AIDS, Vancouver, Canada; 4 Department of Chemistry, Simon Fraser University, Burnaby, Canada; 5 Division of Medical Microbiology and Virology, St Paul’s Hospital, Vancouver, Canada; 6 Department of Medicine, University of British Columbia, Vancouver, Canada; 7 Department of Pathology and Laboratory Medicine, University of British Columbia, Vancouver, Canada; 8 Department of Pathology and Laboratory Medicine, Providence Health Care, Vancouver, Canada

**Keywords:** COVID-19, mRNA vaccine, humoral responses, older adults, antibodies, viral neutralization

## Abstract

**Background:**

The magnitude and durability of immune responses to coronavirus disease 2019 (COVID-19) mRNA vaccines remain incompletely characterized in the elderly.

**Methods:**

Anti-spike receptor-binding domain (RBD) antibodies, angiotensin-converting enzyme 2 (ACE2) competition, and virus neutralizing activities were assessed in plasma from 151 health care workers and older adults (range, 24–98 years of age) 1 month following the first vaccine dose, and 1 and 3 months following the second dose.

**Results:**

Older adults exhibited significantly weaker responses than younger health care workers for all humoral measures evaluated and at all time points tested, except for ACE2 competition activity after 1 vaccine dose. Moreover, older age remained independently associated with weaker responses even after correction for sociodemographic factors, chronic health condition burden, and vaccine-related variables. By 3 months after the second dose, all humoral responses had declined significantly in all participants, and remained significantly lower among older adults, who also displayed reduced binding antibodies and ACE2 competition activity towards the Delta variant.

**Conclusions:**

Humoral responses to COVID-19 mRNA vaccines are significantly weaker in older adults, and antibody-mediated activities in plasma decline universally over time. Older adults may thus remain at elevated risk of infection despite vaccination.

## Teaser

COVID-19 mRNA vaccines induce weaker antibody responses in older adults. Age was a significant predictor of spike binding antibody concentration and SARS-CoV-2 neutralizing activity after correcting for participant demographics, including chronic health conditions.

Older age is the strongest and most common risk factor for lethal coronavirus disease 2019 (COVID-19) following severe acute respiratory syndrome coronavirus 2 (SARS-CoV-2) infection [1–3]. While COVID-19 vaccines offer hope to end the pandemic [4–7], real-world assessments have revealed weaker vaccine-induced immune responses in certain groups including the elderly [8–16], although few studies have adjusted for potential confounders, including comorbidities, that can accumulate with age. Vaccine response durability and cross-reactivity towards SARS-CoV-2 variants of concern also remain incompletely characterized, as immunogenicity assessments are occurring concomitantly with national vaccine rollouts and emergence of new strains.

Two mRNA vaccines, Comirnaty (BNT162b2; Pfizer/BioNTech) and Spikevax (mRNA-1273; Moderna), have been administered widely. Both vaccines provided > 94% protection against moderate or severe COVID-19 in clinical trials after 2 doses [6, 7] and population-level reductions in COVID-19 were observed following initial vaccine rollouts, but ongoing outbreaks in long-term care facilities underscore the continuing vulnerability of older adults to SARS-CoV-2, even after vaccination [14, 17, 18]. Age and age-associated comorbidities, including chronic health conditions that result in immune dysregulation, have been linked to poor vaccine responses [19–21], but few studies have explored these variables in the context of COVID-19 immunization. A better understanding of the impact of age and age-related factors on the magnitude and durability of vaccine-induced immune responses can inform public health decision-making around COVID-19 vaccine allocation as the pandemic progresses.

We investigated the magnitude of SARS-CoV-2 spike-specific humoral immune responses in plasma 1 month after the first mRNA vaccine dose, and 1 and 3 months following the second dose in 151 participants aged 24–98 years. We also assessed responses against the widely circulating Delta variant (B.1.617.2) at 1 and 3 months following the second vaccine dose. Our results demonstrate weaker humoral responses to COVID-19 mRNA vaccines in older versus younger adults, signified by reduced magnitude and durability of spike-specific binding antibodies, ACE2 competition activity, and neutralizing antibody activity even after correction for potential confounders. Reduced humoral responses were also observed against the Delta variant, indicating that older adults may remain at higher risk of infection by this predominant circulating strain despite vaccination.

## METHODS

### Study Design

We conducted a prospective longitudinal cohort study in British Columbia, Canada, to examine SARS-CoV-2–specific humoral immune responses following vaccination with COVID-19 mRNA vaccines Comirnaty (BNT162b2; Pfizer/BioNTech) or Spikevax (mRNA-1273; Moderna). Our cohort of 151 individuals included 89 health care workers and 62 older adults (comprising 23 residents of long-term care or assisted living facilities and 39 seniors living independently).

### Ethics Approval

Written informed consent was obtained from all participants or their authorized decision makers. This study was approved by the University of British Columbia/Providence Health Care and Simon Fraser University Research Ethics Boards.

### Participants and Sampling

Participants were recruited at facilities operated by Providence Health Care (Vancouver, Canada) and from the community. Serum and plasma were collected prior to vaccination, at 1 month after the first dose, and at 1 and 3 months after the second dose. Specimens were processed the same day and frozen until analysis.

### Data Sources and Immune Measures

Sociodemographic data (age, sex, ethnicity), chronic health conditions, and COVID-19 vaccination information were collected by self-report and confirmed through medical records where available. Chronic health conditions were defined as hypertension, diabetes, asthma, obesity (defined as having a body mass index ≥ 30), chronic diseases of lung, liver, kidney, heart or blood, cancer, and immunosuppression due to chronic conditions or medication, to generate a total score ranging from 0 to 11 per participant. Vaccine-induced responses were assessed using (1) a commercial assay to detect immunoglobulin G (IgG) antibodies targeting the spike receptor-binding domain (RBD); (2) a commercial angiotensin-converting enzyme 2 (ACE2) competition assay to detect antibodies that block RBD-receptor interaction; and (3) virus neutralization assays to detect antibodies that prevent SARS-CoV-2 infection of target cells.

#### Binding Antibody Assays

COVID-19–convalescent individuals were identified by the presence of serum antibodies recognizing SARS-CoV-2 nucleoprotein (N) using the Elecsys Anti-SARS-CoV-2 assay on a Cobas e601 module analyzer (Roche Diagnostics). Plasma IgG binding antibodies against RBD were quantified using enzyme-linked immunosorbent assay (ELISA; V-plex SARS-CoV-2; Meso Scale Diagnostics) on a Meso QuickPlex SQ120 instrument as directed by the manufacturer. Results were calibrated against a World Health Organization-referenced standard and are report as international binding antibody units (BAU)/mL.

#### ACE2 Competition Assay

The ability of plasma antibodies to block the RBD-ACE2 receptor interaction was assessed by competition ELISA (V-plex SARS-CoV-2; Meso Scale Diagnostics) on a Meso QuickPlex SQ120 instrument as directed by the manufacturer. Results were calibrated against an external standard and are reported as arbitrary units (AU)/mL, with an upper limit of quantification of 35 (or 1.54 log_2_) AU/mL.

#### Virus Neutralization Assays

Neutralizing activity in plasma was examined using a live SARS-CoV-2 infectivity assay at containment level 3. Assays were performed using isolate USA-WA1/2020 (BEI Resources) and VeroE6-TMPRSS2 (JCRB-1819) target cells. Virus stock was adjusted to 50 TCID_50_/200 µL in Dulbecco’s Modified Eagle Medium in the presence of serial 2-fold dilutions of plasma (1:20 to a maximum of 1:2560), incubated at 4°C for 1 hour, then added to target cells in 96-well plates in triplicate and incubated at 37°C with 5% CO_2_. Viral cytopathic effect (CPE) was recorded on day 3 postinfection. Neutralizing activity is reported as present if CPE was prevented in all 3 wells at a 1:20 dilution (binary variable); or as the reciprocal plasma dilution necessary to prevent CPE in all 3 wells (continuous variable). After 1 dose of vaccine, neutralizing activity was reported as borderline if CPE was prevented in any of 3 wells at a 1:20 dilution.

### Statistical Analysis

Comparisons of binary variables were performed using Fisher exact test. Comparisons of continuous variables were performed using the Mann-Whitney *U* test (for unpaired data) or Wilcoxon test (for paired data). Ordinary least squares regression was used to examine relationships between continuous variables. Multiple linear regression was employed to investigate the relationship between age (per year increment), sex (female as reference group), Ethnicity (nonwhite as reference group), number of chronic health conditions (per number increment), vaccine type (Comirnaty as reference group), dosing interval (per day increment), and sampling date following vaccine dose (per day increment) on immunogenicity outcomes. All tests were 2-tailed, with *P* = .05 considered statistically significant. Analyses were conducted using Prism version 9.3.0 (GraphPad).

## RESULTS

### Lower RBD Binding Antibodies Associated With Older Age and Chronic Health Conditions

Characteristics of the 151 participants, which included 89 health care workers (HCW) and 62 older adults, are shown in Table 1. All participants received 2 doses of mRNA vaccine between December 2020 and July 2021. Due to limited initial vaccine supply in British Columbia, the interval between doses was extended to a maximum of 112 days on 1 March 2021, so participants received their second dose a median of 91 days after the first (interquartile range [IQR], 70–99 days). Samples were collected before vaccination to assess prior exposure to SARS-CoV-2 (n = 142); at 1 month following the first (n = 141) and second (n = 150) doses to quantify response magnitude; and at 3 months following the second dose (n = 150) to examine response durability.

**Table 1.** Study Participants (n = 151)

Characteristic	Health Care Workers (n = 89)	Older Adults (n = 62)	*P* Value
Age, y, median (IQR)^a^	41 (35–50)	79 (73–86)	**<.0001**
Female sex, n (%)	65 (73)	43 (69)	.71
White ethnicity, n (%)	40 (45)	47 (76)	**.0002**
COVID-19 convalescent anti-N Ab^+^, n (%)	8 (9)	6 (10)	>.99
Chronic health or immunosuppressive condition, median (IQR)	0 (0–0)	1 (0–2)	**<.0001**
Comirnaty mRNA vaccine, n (%)	88 (99)	54 (87)	**.0015**
Time between doses, d, median (IQR)	97 (91–103)	78 (45–86)	**<.0001**
Specimens collected 1 mo after first dose, n (%)	87 (98)	54 (87)	NA
Day of specimen collection 1 mo after first dose, median (IQR)	29 (27–31)	30 (28–32)	**.0069**
Specimens collected 1 mo after second dose, n (%)	89 (100)	61 (98)	NA
Day of specimen collection 1 mo after second dose, median (IQR)	30 (29–32)	30 (29–32)	.78
Specimens collected 3 mo after second dose, n (%)	89 (100)	61 (98)	NA
Day of specimen collection 3 mo after second dose, median (IQR)	90 (90–91)	90 (88–91)	**.011**

Statistically significant *P* values (< .05) are highlighted using bold text.

Abbreviations: Ab, antibody; IQR, interquartile range; N, nucleoprotein, NA, not applicable.

As shown in Table 1, HCW and older adults were a median of 41 and 79 years old respectively, and predominantly female. At entry, 14 participants (9.3%; 8 HCW and 6 older adults) were identified as COVID-19–convalescent based on the presence of anti–SARS-CoV-2 N antibodies. Nine participants (6%; 1 HCW and 8 older adults) received Spikevax for their first dose, while 142 (94%) received Comirnaty. In addition to age, the groups differed significantly in terms of ethnicity (*P* = .0002), number of chronic health conditions (*P* < .0001, where the 2 most common conditions were hypertension and diabetes), vaccine received (*P* = .0015), and time between doses (*P* < .0001). The groups also differed in terms of the exact day of specimen collection after the first dose (*P* = .0069) and at 3 months after the second dose (*P* = .011), although these differences were 1 day or less.

**Figure 1.**
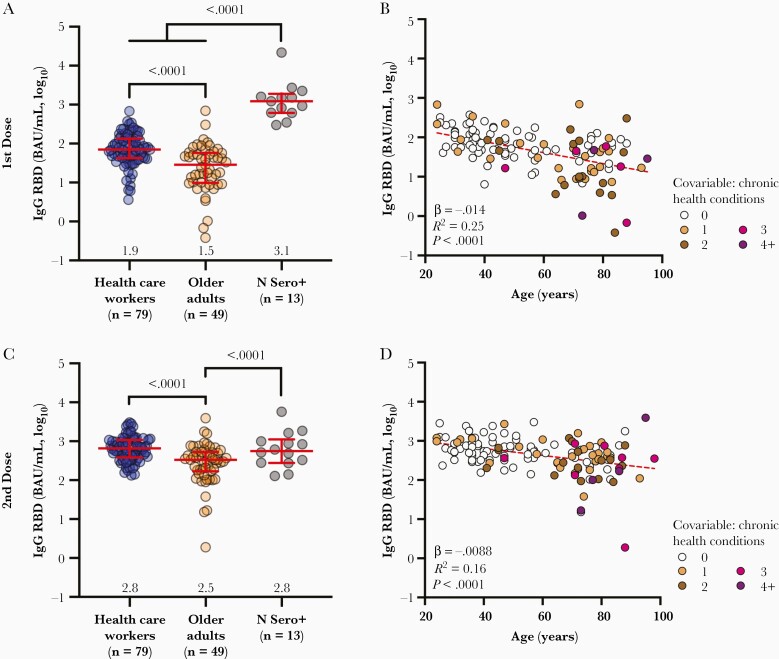
Vaccine-induced binding antibody responses to spike are lower in older adults. *A*, Binding IgG responses to the SARS-CoV-2 spike RBD in plasma, measured by ELISA, following 1 dose of a COVID-19 mRNA vaccine, are shown for health care workers (blue circles) and older adults (orange circles) who were COVID-19 naive at study entry. The third group (N Sero+; grey circles) denotes convalescent participants with anti-N antibodies at study entry. Red bars and whiskers represent median and interquartile range, with group medians shown below. *P* values computed using the Mann-Whitney *U* test are shown above each comparison. *B*, Same data from the COVID-19–naive participants shown in *A* but plotted by age, and where symbols are colored based on the participants’ number of chronic health conditions, which remained significant in multivariable analyses (Table 2). Statistics were computed using ordinary least-squares regression (red dashed line). *C* and *D,* Same as *A* and *B* but for responses measured 1 month following 2 doses of mRNA vaccine. Abbreviations: BAU, binding antibody unit; ELISA, enzyme-linked immunosorbent assay; IgG, immunoglobulin G; N, nucleoprotein; RBD, receptor-binding domain; SARS-CoV-2, severe acute respiratory syndrome coronavirus 2.

We quantified anti-RBD IgG binding antibodies in plasma 1 month after the first and second vaccine doses using ELISA, where the latter time point should capture peak immunity. After 1 dose, median anti-RBD IgG concentrations were 2.5-fold lower in older adults who were naive to COVID-19, compared to COVID-19–naive HCW (*P* < .0001; Figure 1A). In contrast, COVID-19–convalescent participants mounted approximately 17-fold and approximately 42-fold higher IgG responses after 1 dose compared to COVID-19–naive HCW and older adults, respectively (both *P* < .0001), consistent with prior studies demonstrating robust reactivity to 1 dose in previously infected individuals [22, 23]. After 2 doses, median anti-RBD IgG concentrations increased by approximately 10-fold in both naive groups, but responses remained 2-fold lower among older adults (*P* < .0001; Figure 1C). No further increase in IgG antibodies was observed in convalescent participants. Indeed, after 2 doses the median IgG values in HCW reached equivalence with the convalescent group, while values in older adults remained 1.7-fold lower (*P* < .0001; Figure 1C). Of note, 1 doubly vaccinated older adult continued to exhibit a very poor response (Figure 1C).

**Table 2.** Multivariable Analyses

**Immunogenicity Outcome**	**Variable**	**Time Point**					
		1 mo After 1st Dose		1 mo After 2nd Dose		3 mo After 2nd Dose	
		**β Estimate (95% CI)**	***P***	**β Estimate (95% CI)**	***P***	**β Estimate (95% CI)**	***P***
RBD IgG, log_10_ international units	age, per y	−.011 (−.017 to −.0055)	**.0001**	−.0090 (−.014 to −.0044)	**.0002**	−.0080 (−.012 to −.0039)	**.0002**
	Male sex	−.068 (−.25 to .12)	.5	−.11 (−.27 to .053)	.2	.044 (−.097 to .18)	.5
	White ethnicity	−.062 (−.25 to .12)	.5	.063 (−.095 to .22)	.4	.17 (.031 to .31)	**.02**
	No. chronic conditions, per additional	−.10 (−.19 to −.0073)	**.03**	−.047 (−.12 to .028)	.2	−.070 (−.14 to −.0038)	**.04**
	Spikevax vaccine	.26 (−.83 to .60)	.1	.20 (−.059 to .46)	.1	.15 (−.088 to .39)	.2
	Sampling date, per d^^a^^	−.012 (−.047 to .023)	.5	.0040 (−.027 to .035)	.8	.0036 (−.021 to .028)	.8
	Dosing interval, per d^^b^^	NA	…	−.0016 (−.0050 to .0017)	.3	−.0017 (−.0046 to .0013)	.3
ACE2 competition, log_10_ units	Age, per year	−.0016 (−.0040 to .00088)	.2	−.0053 (−.0096 to −.00093)	**.02**	−.0039 (−.0071 to −.00069)	**.02**
	Male sex	−.094 (−.18 to −.011)	**.03**	−.068 (−.22 to .082)	.4	.019 (−.095 to .13)	.7
	White ethnicity	−.046 (−.13 to .035)	.3	−.098 (−.25 to .051)	.2	.064 (−.047 to .17)	.3
	No. chronic conditions, per additional	−.015 (−.056 to .026)	.5	−.029 (−.10 to .042)	.4	−.028 (−.081 to .026)	.3
	Spikevax vaccine	.098 (−.052 to .25)	.2	.12 (−.12 to .37)	.3	.15 (−.049 to .34)	.1
	Sampling date, per d^^a^^	.0064 (−.0091 to .022)	.4	−.0070 (−.036 to .022)	.6	−.000058 (−.0023 to .0024)	> .99
	Dosing interval, per d^^b^^	NA	…	.0019 (−.0012 to .050)	.2	−.0069 (−.027 to .013)	.5
Viral neutralization, log_2_ reciprocal dilution	Age, per y	−.0080 (−.013 to −.0030)	**.002**	−.032 (−.048 to −.016)	**.0002**	−.025 (−.040 to −.0095)	**.002**
	Male sex	−.079 (−.25 to .090)	.4	−.36 (−.92 to .21)	.2	.11 (−.43 to .65)	.7
	White ethnicity	.0078 (−.16 to .17)	.9	−.14 (−.70 to .42)	.6	.25 (−.28 to .78)	.3
	No. chronic conditions, per additional	.049 (−.034 to .13)	.2	−.056 (−.32 to .21)	.7	−.16 (−.41 to .097)	.2
	Spikevax vaccine	.40 (.014 to .79)	**.04**	.75 (−.17 to 1.67)	.1	.38 (−.54 to 1.32)	.4
	Sampling date, per d^^a^^	.0077 (−.024 to .039)	.6	.022 (−.088 to .13)	.7	−.071 (−.17 to .023)	.1
	Dosing interval, per d^^b^^	NA	…	.0024 (−.0094 to .014)	.4	−.00088 (−.012 to .010)	.9

Statistically significant *P* values (< .05) are highlighted using bold text.

Abbreviations: CI, confidence interval; IgG, immunoglobulin G; NA, not applicable; RBD, receptor-binding domain.

Day of specimen collection following last dose.

Days elapsed between first and second vaccine dose (where applicable).

Among COVID-19–naive individuals, we estimated using univariable linear regression that every decade of older age was associated, on average, with 0.14 and 0.09 log_10_ lower IgG responses 1 month after 1 and 2 vaccine doses, respectively (both *P* < .0001; Figure 1B and 1D). Multivariable analyses adjusting for sex, ethnicity, number of chronic health conditions, vaccine brand, dosing interval, and day of specimen collection postimmunization confirmed that older age remained significantly negatively associated with IgG responses 1 month after 1 and 2 vaccine doses (*P* = .0001 and *P* = .0002, respectively). A higher number of chronic health conditions was also negatively associated with IgG responses after 1 dose (*P* = .03; Table 2).

### Reduced Ability to Block ACE2 Binding Associated With Older Age and Male Sex

**Figure 2.**
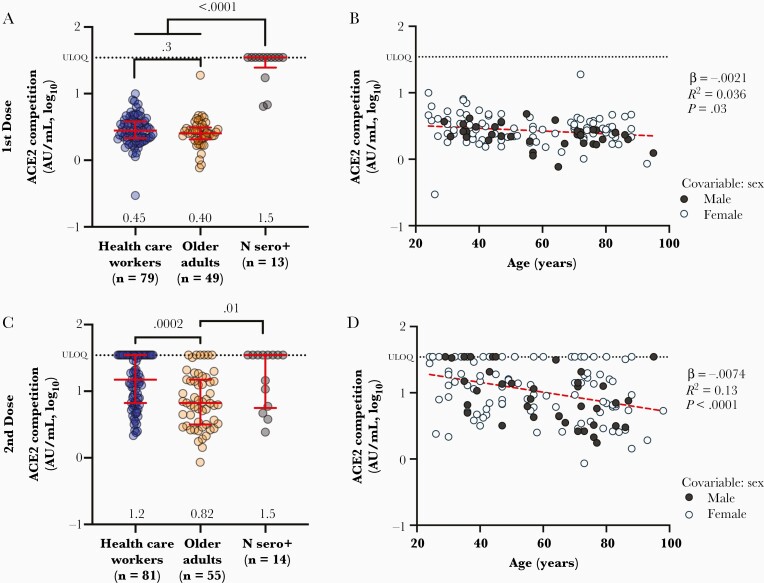
Vaccine-induced ACE2 receptor competition activity is weaker in older adults. *A*, Ability of vaccine-induced plasma antibodies to displace soluble ACE2 receptor from spike RBD, measured by ELISA, following 1 dose of vaccine in COVID-19–naive health care workers (blue circles) and older adults (orange circles), as well as COVID-19–convalescent participants (N Sero+; grey circles). Values represent AU/mL calibrated against an external standard, reported in log_10_ units. Red bars and whiskers represent median and interquartile range, with group medians shown below. *P* values computed using the Mann-Whitney *U* test are shown above each comparison. *B*, Same data from the COVID-19–naive participants shown in *A* but plotted by age, and colored by sex, which remained significant in multivariable analyses following 1 dose (Table 2). Statistics were computed using ordinary least-squares regression (red dashed line). *C* and *D,* Same as *A* and *B* but for responses measured 1 month following 2 doses of mRNA vaccine. Abbreviations: ACE2, angiotensin-converting enzyme 2; AU, arbitrary unit; COVID-19, coronavirus disease 2019; ELISA, enzyme-linked immunosorbent assay; RBD, receptor-binding domain; ULOQ, upper limit of quantification.

We next assessed the ability of plasma antibodies to block the interaction between RBD and ACE2 receptor using competition ELISA, which offers a surrogate measure of virus neutralizing activity [24]. After 1 vaccine dose, HCW and older adults exhibited median ACE2 competition activities of 2.8 (or 0.45 log_10_) and 2.5 (or 0.40 log_10_) AU/mL, respectively, a difference that was not statistically significant (Figure 2A). In contrast, after 1 dose most (10, 77%) convalescent participants exhibited a median activity above the upper limit of quantification (ULOQ) for this assay (35, or 1.54 log_10_ AU/mL) (*P* < .0001 compared to both naive groups). One month following the second vaccine dose, HCW exhibited a median activity of 15 (or 1.2 log_10_) AU/mL compared to 6.7 (or 0.82 log_10_) AU/mL in older adults (*P* = .0002; Figure 2C), with 26 (44%) HCW and 6 (11%) older adults above the ULOQ. Meanwhile, convalescent participants maintained a median activity of 35 (1.54 log_10_) AU/mL after the second dose, with 8 (57%) individuals exceeding the ULOQ (*P* = .0006 compared to older adults). These results are consistent with other studies showing that qualitative features of antibody function including virus neutralizing activity may be enhanced following infection compared to vaccination [25, 26], and further suggest that these features may be diminished with older age.

Even though ACE2 competition activities were not significantly different between HCW and older adults following 1 vaccine dose, age-related effects were apparent when age was analyzed as a continuous variable in all COVID-19–naive participants. Specifically, we estimated using univariable linear regression that every 10 years of older age was associated with 0.021 and 0.071 log_10_ AU/mL lower ACE2 competition activity (equivalent to 1.0 and 1.2 AU/mL) 1 month after the first and second doses, respectively (*P* = .03 and < .0001; Figure 2B and 2D). Multivariable analyses confirmed that age remained negatively associated with ACE2 competition activity 1 month after the second dose (*P* = .02; Table 2). Female sex was independently associated with 0.094 log_10_ (or 1.24) AU/mL higher ACE2 competition activity after the first dose (*P* = .03), which is consistent with reports that women display higher neutralizing responses following infection and vaccination [27].

### Weaker Virus Neutralizing Activity Associated With Age and Vaccine Product

**Figure 3.**
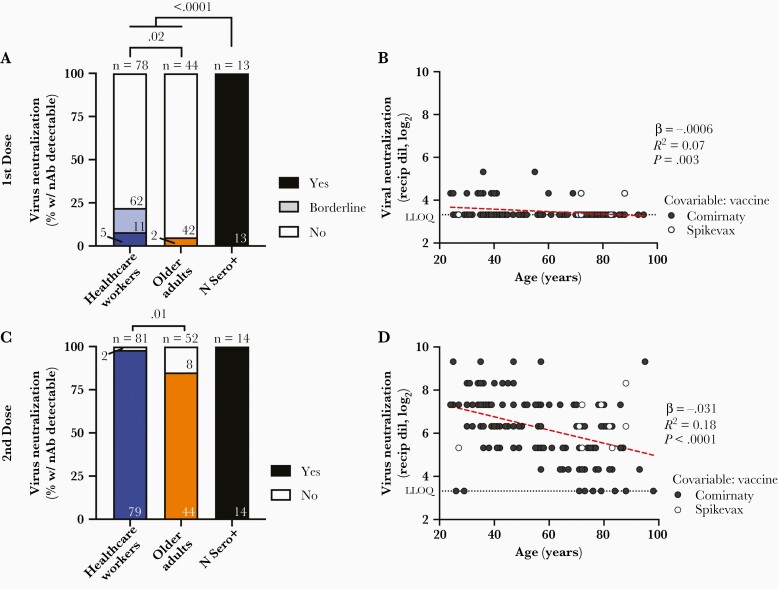
Viral neutralization activity of vaccine-induced antibodies is weaker in older adults. *A*, Frequency of COVID-19–naive health care workers (blue) and older adults (orange), as well as convalescent participants (N Sero+; black) are shown as histograms. The proportion of participants in each group who displayed neutralizing activity against live SARS-CoV-2 (USA-WA1/2020 strain) in 3/3 wells at a 1:20 or higher plasma dilution is shown in the darkest color, while those displaying borderline activity (defined as neutralization in least 1 well at a 1:20 dilution) is shown in lighter color. Total number in each group is shown above each bar, with the number of participants displaying each activity shown within the bar. *P* values computed using Fisher exact test are shown above each comparison. *B*, Same data as the COVID-19 participants shown in *A* (where neutralization includes both yes and borderline categories) but plotted by age, and where neutralization is reported as the reciprocal log_2_ dilution value. Samples that displayed no evidence of neutralization were coded as having a reciprocal dilution factor of 10 (3.32 log_2_). Symbols are colored by vaccine received, which was significantly associated with neutralization activity after 1 dose (Table 2). Statistics were computed using ordinary least-squares regression. *C* and *D*, Same as *A* and *B* but for neutralization responses measured 1 month after 2 doses of mRNA vaccine and where neutralizing activity required inhibition of cytopathic effects in 3/3 wells at a 1:20 or higher plasma dilution. Abbreviations: COVID-19, coronavirus disease 2019; LLOQ, lower limit of quantification; Recip dil, reciprocal dilution; SARS-CoV-2, severe acute respiratory syndrome coronavirus 2; w/ nAb, with neutralizing antibodies.

We next performed live SARS-CoV-2 neutralization assays to quantify the ability of plasma to block infection of target cells, which may involve spike epitopes located outside the RBD [28, 29]. As neutralization activities following 1 vaccine dose were generally weak in COVID-19–naive individuals, for this time point we considered both clear positive samples that neutralized virus in all 3 wells and borderline samples that neutralized virus in at least 1 well at a 1:20 dilution. Using this latter definition, 16/78 (21%) HCW and 2/44 (4.8%) older adults displayed evidence of neutralizing activity (*P* = .02; Figure 3A). In contrast, plasma from all 13 convalescent participants neutralized SARS-CoV-2 following 1 vaccine dose (median reciprocal titer of 240). One month following the second vaccine dose and using the more stringent definition of clear positive, 79/81 (98%) HCW displayed neutralizing activity compared to 44/52 (85%) older adults (*P* = .01; Figure 3C).

Among COVID-19–naive individuals, univariable linear regression confirmed a statistically significant inverse relationship between virus neutralization activity and older age following 1 and 2 vaccine doses (*P* = .003 and *P* < .0001, respectively; Figure 3B and 3D). In multivariable analyses, older age remained significantly associated with weaker neutralization activity after both 1 and 2 doses (*P* = .002 and *P* = .0002, respectively; Table 2). Having received Spikevax was also associated with stronger neutralization activity following 1 dose (*P* = .04). Notably, ACE2 competition activity correlated with virus neutralizing activity after the second dose (Spearman ρ ≥ 0.7; all *P* < .0001; Supplementary Figure 1).

### Vaccine-Induced Antibody Responses Decline Over Time in All Ages

**Figure 4.**
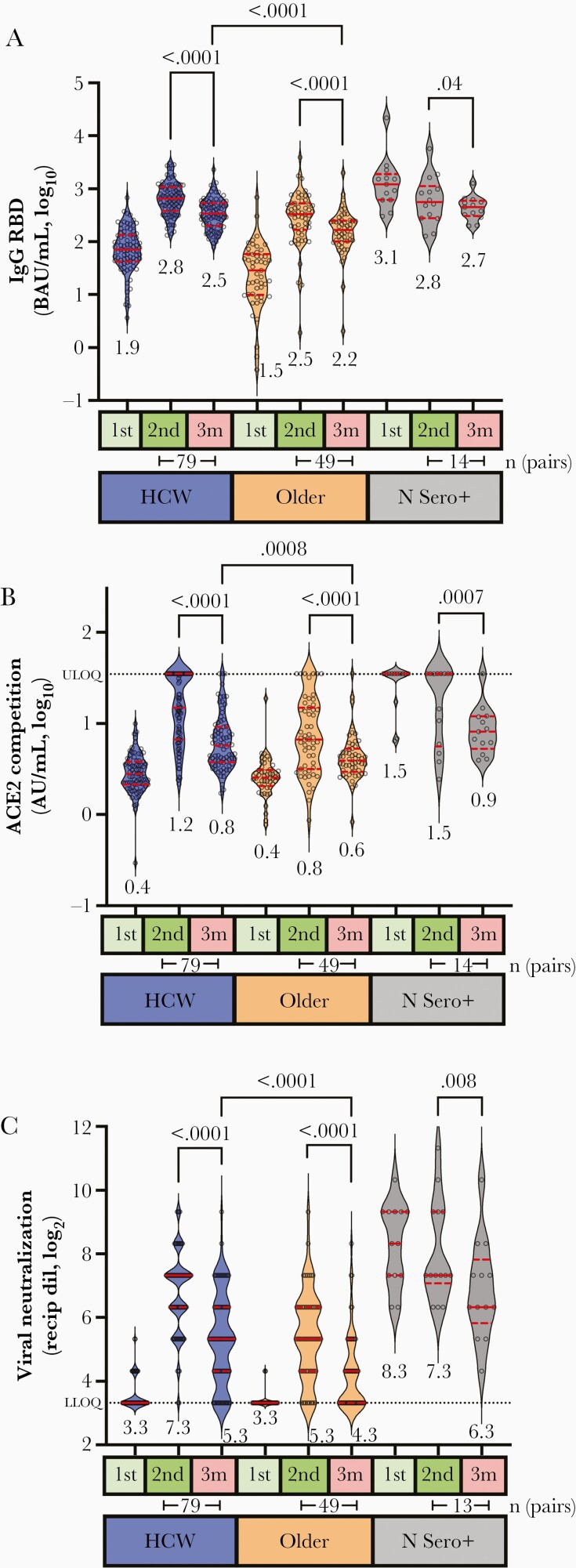
Older adults display lower magnitude and durability of antibody responses after vaccination. *A,* Binding IgG responses to spike RBD, measured by ELISA, 1 month after the first vaccine dose (1st, light green box), 1 month after the second vaccine dose (2nd, green box) and 3 months after this dose (3m, red box) are shown for HCW (blue) and older adults (orange) who were COVID-19–naive at study entry, and convalescent participants (N Sero+). Median values are displayed below each group. *P* values (computed using the Wilcoxon paired test for within-group comparisons, and the Mann-Whitney *U* test for between-group comparisons) are shown above each comparison performed. The number of pairs compared in the Wilcoxon paired test is shown below the relevant time points. *B*, ACE2 competition assay results in the same individuals, measured by ELISA. Values represent AU/mL calibrated against an external standard, reported in log_10_ units*. C*, Virus neutralization assay results in the same individuals, displayed as the reciprocal log_2_ plasma dilution. Note that some values are superimposed. Abbreviations: ACE2, angiotensin-converting enzyme 2; AU, arbitrary unit; BAU, binding antibody units; COVID-19, coronavirus disease 2019; ELISA, enzyme-linked immunosorbent assay; HCW, health care worker; IgG, immunoglobulin G; LLOQ, lower limit of quantification; RBD, receptor-binding domain; Recip dil, reciprocal dilution; ULOQ, upper limit of quantification.

To examine immune response durability, we reassessed humoral outcomes 3 months following the second vaccine dose. All 3 measures of antibody activity declined between 1 and 3 months following the second immunization: median IgG binding antibodies declined 2-fold in both HCW and older adults (Wilcoxon paired test, both *P* < .0001; Figure 4A), while median ACE2 competition activity declined by 2.6-fold in HCW and by 1.7-fold in older adults (Wilcoxon, both *P* < .0001; Figure 4B). Furthermore, median virus neutralizing activity declined 4-fold in HCW (*P* < .0001) and 2-fold in older adults (Wilcoxon, *P* < .0001; Figure 4C). Despite these temporal reductions, responses in HCW remained significantly higher compared to older adults in all assays at 3 months after the second dose (Supplementary Figure 2), and age remained a significant independent predictor of reduced activity for all measures in both univariable and multivariable analyses (Supplementary Figure 2 and Table 2). For context, the median residual activities observed in HCW at 3 months after the second dose were comparable to peak responses seen in older adults at 1 month after this dose.

### Impaired Ability to Block ACE2 Binding by Delta Variant Among Older Adults

**Figure 5.**
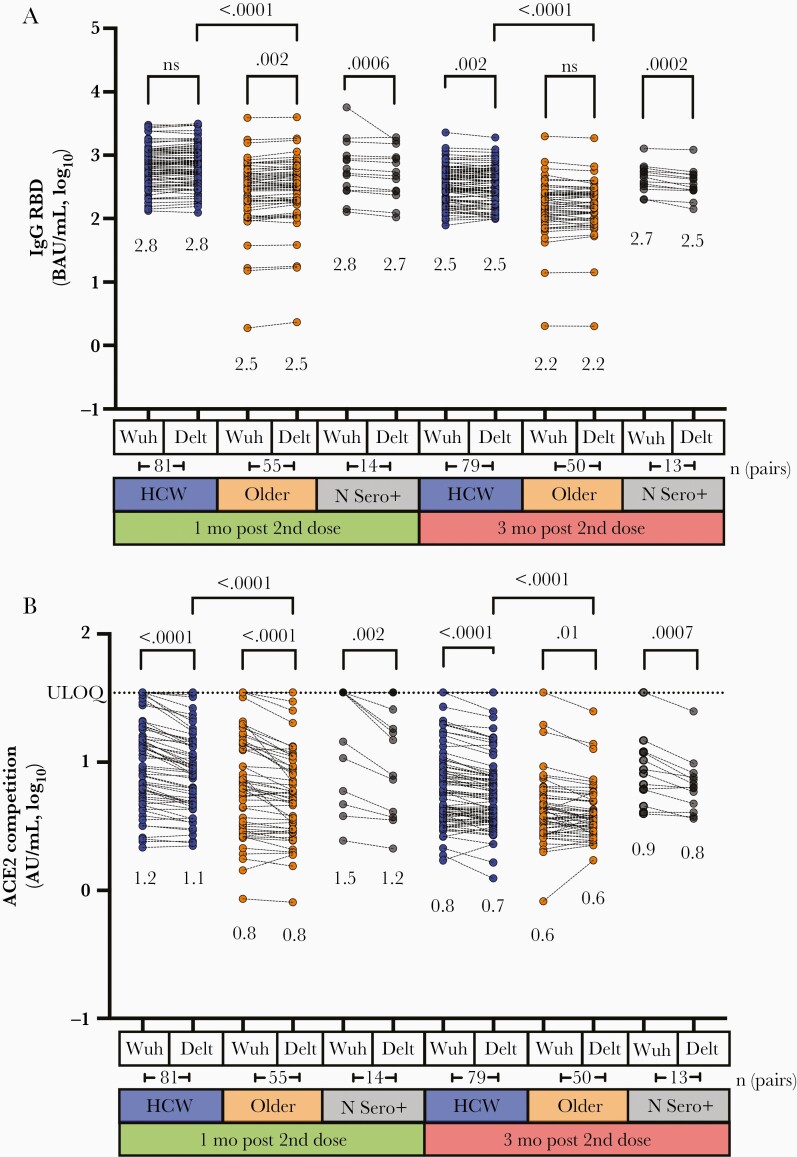
Cross-reactive antibody responses against the Delta variant after 2 doses of mRNA vaccine. *A,* Binding IgG antibody responses to spike RBD from the original Wuhan strain and the Delta variant, measured by ELISA, 1 month following the second vaccine dose (peak; green) and 3 months after this dose (durability; red) in HCW (blue) and older adults (orange) who were COVID-19 naive at study entry, and convalescent participants (N Sero+; grey). Median values are shown below each group. *P* values (computed using the Wilcoxon paired test for within-group comparisons, and the Mann-Whitney *U* test for between-group comparisons) are shown above each comparison performed. The number of pairs compared in the Wilcoxon paired test is shown below the relevant time points. *B*, ACE2 competition results in the same individuals, measured by ELISA. Values represent AU/mL calibrated against an external standard, reported in log_10_ units. Abbreviations: ACE2, angiotensin-converting enzyme 2; AU, arbitrary unit; BAU, binding antibody units; COVID-19, coronavirus disease 2019; Delt, Delta; ELISA, enzyme-linked immunosorbent assay; HCW, health care worker; ns, not significant; RBD, receptor-binding domain; ULOQ, upper limit of quantification; Wuh, Wuhan.

Given concerns that SARS-CoV-2 variants may be more transmissible or evade aspects of host immunity [30–32], we examined IgG binding antibodies and ACE2 competition activity against the B.1.617.2 (Delta) variant at 1 and 3 months following the second vaccine dose. Consistent with our observations for the original Wuhan strain, binding antibody responses to the Delta RBD were approximately 2-fold lower among older adults compared to HCW at both time points (Mann-Whitney, both *P* < .0001; Figure 5A). Within each group however, median binding antibody values were broadly comparable between the 2 strains at each time point tested, where within-group comparisons were either not significantly different or only modestly lower despite achieving statistical significance (eg, differences < 0.02 log_10_ for HCW and older adults; <0.08 log_10_ for convalescents). ACE2 competition activity against Delta RBD was also significantly lower among older adults compared to HCW at 1 and 3 months after the second dose (Mann-Whitney, both *P* < .0001; Figure 5B). Moreover, plasma specimens from all groups consistently displayed significantly weaker ability to block ACE2 receptor engagement by the Delta RBD compared to that of Wuhan RBD (Wilcoxon, all *P* ≤ .01), although the magnitude of these differences was modest (approximately 0.04 log_10_ in HCW, approximately 0.03–0.06 log_10_ in older adults).

## DISCUSSION

This study extends our understanding of antibody response magnitude and durability following COVID-19 mRNA vaccination across the adult age spectrum [14, 18, 33–36]. Overall, responses in older adults are impaired both quantitatively (ie, fewer binding antibodies) and functionally (ie, lower ACE2 displacement and neutralization activities) compared to younger adults, even after 2 vaccine doses. Importantly, multivariable analyses confirmed older age as an independent determinant of poorer immune responses at nearly all time points evaluated following both 1 and 2 vaccine doses, even after controlling for chronic health conditions that can accumulate with age and compromise immunity [19–21]. The sole exception was ACE2 competition activity 1 month after the first dose, which did not remain independently associated with age after multivariable correction. Multivariable analyses identified additional correlates of humoral responses following the first vaccine dose, including the number of chronic health conditions (associated with lower binding antibody titers), male sex (associated with lower ACE2 competition activity), and having received Spikevax (associated with higher virus neutralizing activity). In general, the impact of these variables on humoral responses diminished after the second vaccine dose, although the number of chronic conditions was again associated with poorer binding antibody responses 3 months after the second dose. Our results thus identify age as the most critical and consistent variable modulating the magnitude of antibody responses after COVID-19 mRNA vaccination.

Our findings also shed light on the short-term durability of humoral responses to COVID-19 mRNA vaccines. By 3 months following the second vaccine dose, plasma antibody concentrations had declined significantly in all participants, particularly those who were naive to COVID-19 prior to vaccination. Assuming exponential decay, we estimate the half-life of anti-RBD binding antibodies to be 87 days (95% confidence interval 75–97) in the naive group, which suggests that antibody durability following mRNA vaccination may be lower compared to that following infection, which was calculated to be approximately 116 days in a study of convalescent individuals [37]. More importantly, humoral responses remained substantially lower among older adults at all time points tested. For context, the diminished responses observed in HCW at 3 months following the second vaccine dose were comparable to peak levels observed in older adults at 1 month following the second dose. Similar results for antibody binding and ACE2 competition activity were found for the B.1.617.2 (Delta) variant RBD, suggesting that older adults will remain more susceptible to infection by this variant at all stages after vaccination due to their weaker overall responses.

Our observations are consistent with poorer immune responses to certain immunizations (eg, influenza) among older adults that can be mitigated in part by modifying vaccine formulations (eg, by increasing antigen concentrations or additional adjuvants) or providing booster immunizations more frequently [19–21]. Reports from the United Kingdom [15] and Germany [16] have also demonstrated age-related impairments in binding and neutralizing antibodies following immunization with the Comirnaty vaccine, although T-cell responses were more similar between younger and older participants. However, these studies did not examine the durability of vaccine-induced immune responses in older adults, which is of paramount importance as more time elapses after people complete the standard 2-dose vaccine schedule. Indeed, recent increases in SARS-CoV-2 infections among doubly vaccinated individuals [38], including outbreaks in long-term care facilities [17], underscore this ongoing risk.

Our findings that 14% of older adults failed to neutralize SARS-CoV-2 (USA-WA1/2020 strain) 1 month after having received 2 vaccine doses, a time point that should capture the peak vaccine immune response, and that this percentage increased to 44% just 2 months later, further emphasizes the ongoing infection risk in this population. While we did not perform virus neutralization assays using the Delta variant, our ACE2 competition results using the RBD of this strain suggest that neutralization activity against Delta is likely to be lower than that against the Wuhan strain. Given the ability of SARS-CoV-2 variants to evade at least some aspects of vaccine-elicited immunity [39, 40], our results support ongoing prioritization of older adults for receipt of additional vaccine doses.

A limitation of our study is that immune correlates of protection for SARS-CoV-2 transmission and disease severity remain incompletely characterized [41], so the implications of our results as they relate to individual-level control of COVID-19 remain uncertain. Because precise antibody concentrations and activities needed to achieve protection are unknown, it is possible that the vaccine-induced immune responses seen in older adults will be sufficient to prevent symptomatic infection or severe disease in many cases. Additional studies linking vaccine immunogenicity data to clinical outcomes specifically among older adults are needed. In addition, we have not assessed the cellular immune responses induced by vaccination. Antiviral T-cell responses are durable following infection and immunization with mRNA vaccines [42–47] but more research is needed to define their role in long-term protection against infection and disease. Furthermore, due to the small number of participants who received Spikevax, we had low power to assess differences in responses between mRNA vaccines. Nevertheless, and consistent with recent studies [17, 48], Spikevax was associated with improved virus neutralization activity following a single vaccine dose in our analysis.

Overall, our results extend a growing body of evidence indicating that COVID-19 mRNA vaccines are less immunogenic in older adults and further reveal substantial declines of humoral responses in plasma across all ages in the first 3 months following completion of a 2-dose vaccine series. The combined effects of lower peak immunity and natural declines in vaccine-induced humoral responses may leave older adults at continued risk of infection by SARS-CoV-2 or its variants.

## Supplementary Data

Supplementary materials are available at *The Journal of Infectious Diseases* online. Supplementary materials consist of data provided by the author that are published to benefit the reader. The posted materials are not copyedited. The contents of all supplementary data are the sole responsibility of the authors. Questions or messages regarding errors should be addressed to the author.

jiab592_suppl_Supplementary_Figure_S1

jiab592_suppl_Supplementary_Figure_S2

## Notes

***Acknowledgments.*** We thank the leadership and staff of Providence Health Care, including long-term care and assisted living residences, for their support of this study. We thank the phlebotomists and laboratory staff at St Paul’s Hospital, the BC Centre for Excellence in HIV/AIDS, and Simon Fraser University for assistance. Above all, we thank the participants, without whom this study would not have been possible.

***Disclaimer***. The views expressed in this publication are those of the authors and not necessarily those of African Academy of Sciences (AAS), New Partnership for Africa’s Development Planning and Coordinating Agency (NEPAD), Wellcome Trust, or the UK Government.

***Financial support.*** This work was supported by the Public Health Agency of Canada (grant number 2021-HQ-000120 COVID-19 Immunology Task Force COVID-19 Hot Spots Award to M. A. B., Z. L. B., and M. G. R.); the Canada Foundation for Innovation (Exceptional Opportunities Fund—COVID-19 award to M. A. B., M. N., M. L. D., R. P., and Z. L. B.); and the National Institute of Allergy and Infectious Diseases, National Institutes of Health (grant number R01AI134229 to R. P.). G. U. and F. H. O. are supported by PhD fellowships from the Sub-Saharan African Network for TB/HIV Research Excellence, a DELTAS Africa Initiative (grant number DEL-15-006). The DELTAS Africa Initiative is an independent funding scheme of the AAS Alliance for Accelerating Excellence in Science in Africa and supported by the NEPAD Agency with funding from the Wellcome Trust (grant number 107752/Z/15/Z) and the UK Government. L. Y. L. was supported by a Simon Fraser University Undergraduate Research Award. M. L. D. and Z. L. B. hold Scholar Awards from the Michael Smith Foundation for Health Research.

***Potential conflicts of interest.*** All authors: No reported conflicts of interest. All authors have submitted the ICMJE Form for Disclosure of Potential Conflicts of Interest. Conflicts that the editors consider relevant to the content of the manuscript have been disclosed.

Presented in part: 2021 Canadian Association for HIV Research Conference, 5–7 May 2021, virtual, Canada.
