# Supplementary figures and images for: Reduced Magnitude and Durability of Humoral Immune Responses to COVID-19 mRNA Vaccines Among Older Adults
Source: J Infect Dis. 2021 Dec 9;225(7):1129–40. doi: 10.1093/infdis/jiab592 (PMC8689804; doi:10.1093/infdis/jiab592)

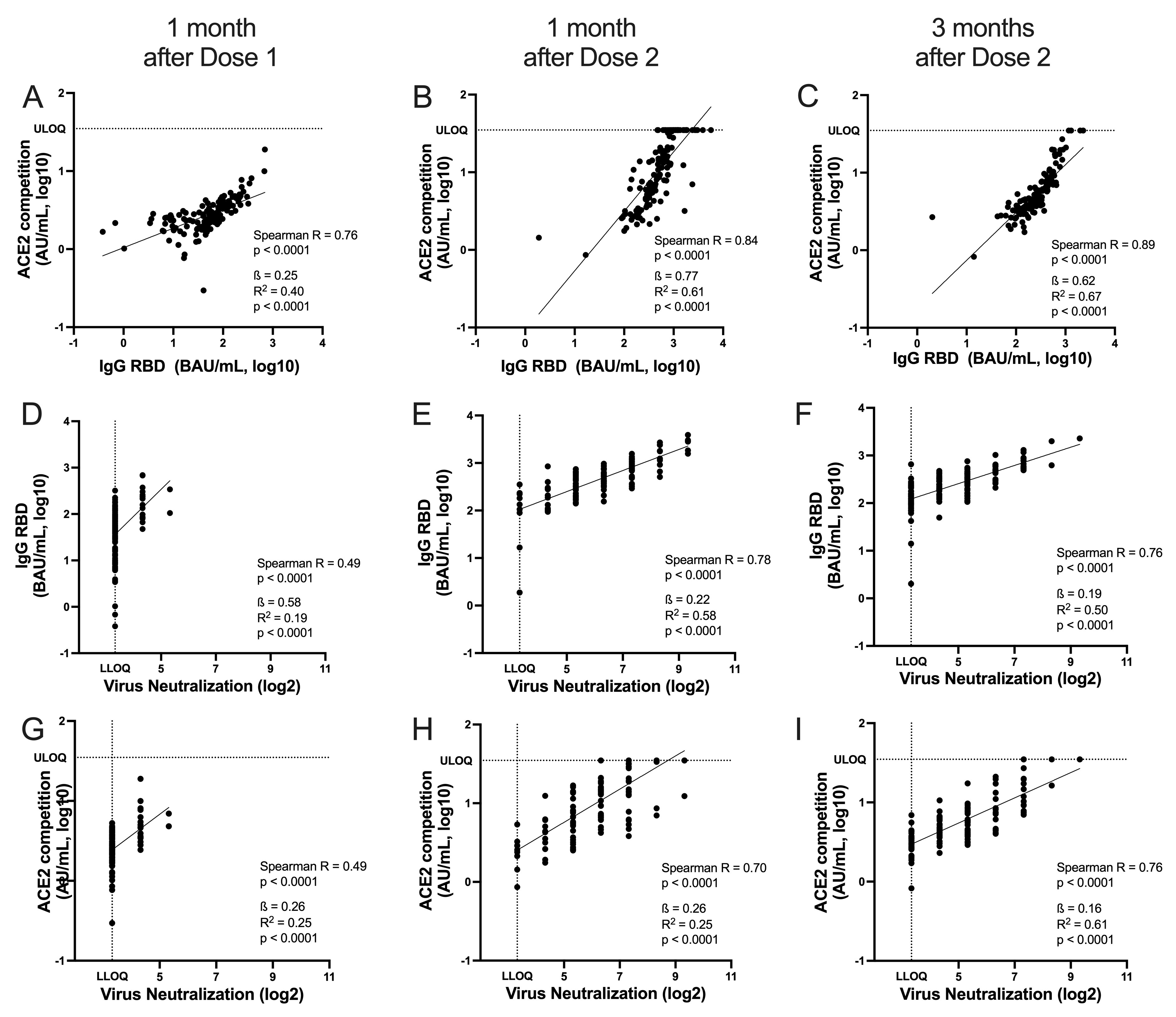

Supplement: jiab592_suppl_Supplementary_Figure_S1 [file jiab592_suppl_supplementary_figure_s1.jpeg]

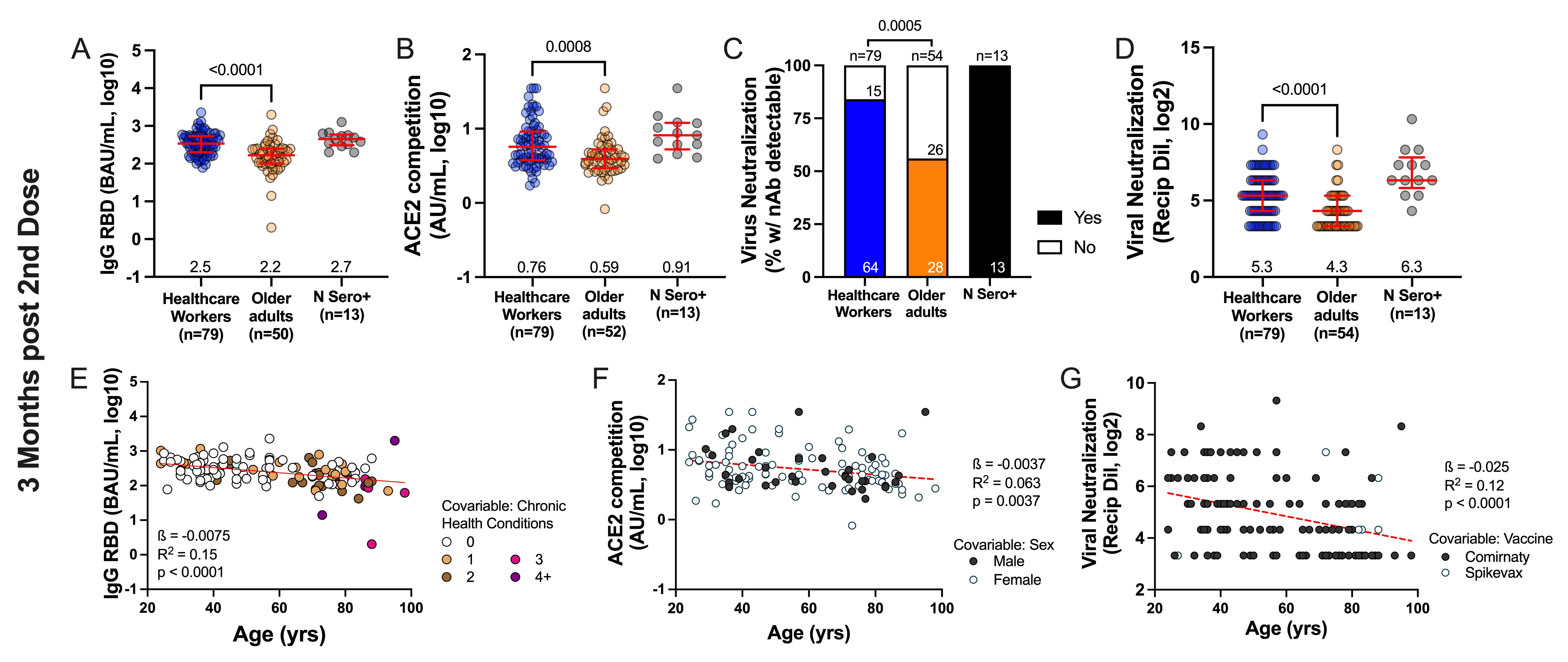

Supplement: jiab592_suppl_Supplementary_Figure_S2 [file jiab592_suppl_supplementary_figure_s2.jpeg]
